# Evaluation of YouTube videos addressing thoracoscopic sympathectomy using the LAP-VEGaS guidelines

**DOI:** 10.3389/fsurg.2023.1133124

**Published:** 2023-03-20

**Authors:** Ottavio Adorisio, Enrico Davoli, Francesco De Peppo

**Affiliations:** ^1^ Department of Pediatric Surgery, Pediatric Surgery Unit, Bambino Gesù Children’s Hospital, IRCCS, Rome, Italy; ^2^ Department of General Surgery, Campus Biomedico University Hospital, Rome, Italy

**Keywords:** thoracoscopic sympathectomy, hyperhidrosis, YouTube®, LAP-VEGaS, internet

## Abstract

**Introduction:**

The study aims to evaluate the quality of videos addressing thoracoscopic sympathectomy on YouTube® using the LAParoscopic surgery Video Educational GuidelineS (LAP-VEGaS) criteria.

**Methods:**

YouTube was searched using the following keyword: “thoracoscopic sympathectomy” on August 22, 2021. The first 50 videos were analyzed and classified for baseline characteristics and conformity to the LAP-VEGaS checklist.

**Results:**

Duration ranged from 19 s to 22 min. The mean number of likes was 14.8 (range 0–80). The mean number of dislikes was 2.5 (range 0–14). The mean number of comments was 8.5 (range 0–67). Nineteen videos did not meet our criteria and were excluded. Regarding the remaining 31 videos, none contained all 16 points of the LAP-VEGaS essential checklist (mean 5.4 points, range 2–14 points), with almost all neglecting preoperative information and outcomes. The mean percentage of conformity was 37% (range 12%–93%). The most viewed videos were not associated with higher conformity to LAP-VEGaS criteria showing only 4/16 points (25%).

**Conclusions:**

The quality of videos addressing TS on YouTube®, based on the LAP-VEGaS checklist may be considered not acceptable. Experienced surgeons and surgeons in trainees should be aware of this when using it as a learning resource in their clinical practice.

## Introduction

Primary palmar hyperhidrosis is a disease of the autonomous nervous system characterized by excessive sweating of the hands that may impair daily activities (1). In general, this can lead to handwriting difficulties, and difficulties to manage a smartphone or a personal computer. It also can cause social isolation and difficulty in playing sports because a ball or other equipment may slip when gripped with a wet hand, especially in children. In the last few years, video-assisted thoracoscopic sympathectomy (VATS) has become the most reliable procedure for the definitive treatment of primary palmar hyperhidrosis (PPH). At the same time, an increasing number of videos addressing these procedures are available on YouTube®. YouTube® is a widely used open-access video-sharing website that allows one to watch a high number of videos and upload an infinitive number of videclips (2). Users can post comments, like, dislike and express their opinion or feelings. The growth, in the last years, of social media platforms, has expanded the access to visual learning tools by surgeons, who can learn how to perform several surgical techniques in which they have no or less experience (3). The main problem of these platforms is the variability of authorship and the nearly total absence of a peer-review process (4). YouTube® is most frequently used as an educational video source for medical students, surgical trainees, and surgeons, with the great part of them using it as a resource for surgical preparation, even if a standard method of evaluating medical videos available on it has not yet been established. The LAP-VEGaS (Laparoscopic surgery Video Educational Guidelines) guidelines were created in 2018 by a panel of thirty-three international members from various surgical subspecialties (5). The main goal of these guidelines is to provide universally accepted criteria applicable to surgical videos uploaded for educational purposes. Sixteen essential criteria were obtained starting from thirty-seven consensus statements. The LAP-VEGaS guidelines have been validated independently as an accurate tool to identify overall high-quality videos suitable for publication or educational purposes (6, 7). The study aims to assess the quality of YouTube Videos addressing VATS evaluating their adherence to the essential LAP-VEGaS guidelines.

## Materials and methods

We performed a search on YouTube® by using the following keyword: “thoracoscopic sympathectomy” and “video-assisted thoracoscopic sympathectomy” on August 22, 2021. The first 50 videos were analyzed, assuming that users rarely extend their search over the first 5 pages (8). Cartoons, schematized videos, promotional/commercial videos, duplicated videos, and videos not in English were excluded (Table 1). The remaining videos were analyzed for baseline characteristics, educational content, and conformity to the LAP-VEGaS guidelines. These guidelines address 16 essential criteria covering five principal domains: video introduction, case presentation, procedures, outcomes and educational content (Table 2). The search for videos was done based on the website's default settings in order of the proposed relevance. The upload day, the running time, the number of views, comments, and likes/dislikes were recorded. The analysis of the videos was performed separately by two of the authors, any discrepancy was resolved by the judgment of the last author reaching a unanimous consensus, blinding respect to the number of views, comments, likes and dislikes.

**Table 1:** Videos analyzed and main characteristics.

Rank	Video Title	Views	Upload date	Lenght (min)	Comments	N° Likes	N° Dislikes
1	ETS (Endoscopic Thoracic Sympathectomy)	24,086	12/11/2011	01:58	44	41	14
2	Endoscopic Thoracic Sympathectomy (ETS) Surgery Patient Review	20,652	01/01/2012	04:06	0	13	13
3	Bilateral Thoracoscopic Sympathectomy For Palmar Hyperhidrosis	15,113	11/07/2012	05:29	9	10	3
4	Endoscopic Thoracic Sympathectomy (ETS)	14,973	01/05/2014	01:45	67	56	10
5	Robotic Bilateral Sympathectomy for Hyperhidrosis	13,584	05/02/2020	16:42	53	80	9
6	Endoscopic Thoracic Sympathectomy (ETS) using titanium clip	10,694	01/01/2012	02:51	7	2	6
7	VATS Sympathectomy for Palmar Hyperhidrosis—Dr Atul Mishra	7,348	09/05/2019	10:05	48	68	6
8	Sympathectomy for Hyperhidrosis	6,476	08/01/2018	02:37	4	57	0
9	Thoracoscopic Sympathectomy Lecture by Dr R K Mishra	5,534	15/02/2017	16:21	11	67	1
10	Hyperhidrosis-Neri Cohen, MD, PhD, GBMC	4,969	09/08/2010	05:36	1	25	4
11	Laparoscopic (R) Sympathectomy	3,054	18/12/2009	05:31	4	3	2
12	Bilateral thoracoscopic sympathetic block by clipping	2,286	28/11/2019	4.17	0	8	1
13	Thoracoscopic sympathectomy for hyperhidrosis	2,027	21/05/2012	00:19	5	1	1
14	Thoracoscopic Sympathectomy	1,875	12/06/2014	02:15	1	4	1
15	Endoscopic Thotacic sSmpathectomy by Needle Scope	1,772	02/12/2013	07:46	0	2	3
16	Exploring surgical outcomes of R4-5 thoracoscopic sympathectomy	1,263	02/07/2018	07:31	0	6	0
17	Master Class on Thoracoscopic Sympathectomy by Dr.R.K.Mishra	1,243	23/01/2014	22:16	0	3	0
18	Thoracoscopic sympathectomy for hyperhidrosis!	630	28/04/2014	02:50	0	1	1
19	Thoracoscopic sympathectomy for hyperhidrosis	499	28/04/2014	01:07	0	0	1
20	Thoracoscopic Sympathectomy	311	29/01/2015	01:58	0	0	0
21	MMCTS—VATS sympathectomy for hyperhidrosis	294	23/11/2020	06:35	0	0	0
22	Endoscopic thotacic sympathectomy	257	11/03/2018	15:48	0	0	0
23	Thoracoscopic sympathectomy	239	17/10/2013	02:06	0	0	0
24	Awake Bilateral Uniportal Video Assisted Thoracoscpic (NIUVATS) Sympathectomy 0.5 cm Incision	232	08/10/2019	01:11	0	6	0
25	Bilateral Thoracoscopic Sympathectomy For Hyperhidrosis by Dr Christophoros Kotoulas	231	11/01/2000	03:23	5	0	0
26	Endoscopic Thoracic Sympathectomy—Assoc. Prof. Erkan Yıldırım	176	31/07/2018	01:28	1	1	1
27	Thoracoscopic sympathectomy	135	14/11/2014	03:21	2	0	0
28	Asvide: Right sided endoscopic thoracic sympathectomy (ETS) at R3 level for palmar hypehidrosis	91	29/04/2020	00:37	0	2	0
29	Endoscopic thoracic sympathectomy (ETS) for palmar hypehidrosis	62	18/003/2019	01:28	0	1	0
30	Thoracoscopic Sympathectomy for palmar Hyperhidrosis	57	06/10/2020	05:49	2	0	0
31	Endoscopic Thoracic Sympathectomy	37	24/07/2019	00:19	0	0	1

**Table 2:** Conformity to LAP-VEGaS checklist for each video.

Video Number	1	2	3	4	5	6	7	8	9	10	11	12	13	14	15	16	17	18	19	20	21	22	23	24	25	26	27	28	29	30	31	Tot	%
Title including pathology and procedure	1	1	1	1	1	1	1	1	1	1	1	1	1	1	0	1	1	1	0	1	1	1	1	1	1	1	1	1	1	1	1	29	96
Authors’ information and disclosures	1	0	0	1	1	0	0	1	1	1	1	0	0	1	1	1	1	0	0	0	1	0	1	1	1	1	0	1	1	0	0	18	58
Patient anonymity	1	1	1	0	1	1	1	1	1	1	1	1	1	1	1	1	1	0	1	1	1	1	1	1	1	1	1	1	1	1	1	29	96
Imaging	0	0	0	0	0	0	0	0	1	1	0	0	0	0	0	1	1	0	0	0	0	0	0	1	0	0	0	0	0	0	0	5	16
Baseline patient characteristics	0	0	0	1	1	0	0	0	0	1	0	0	0	0	0	1	0	0	0	0	1	0	0	0	0	1	0	0	0	0	0	6	19
Pre-operative workup and treatments	0	0	0	0	0	0	0	0	1	1	0	0	0	0	0	1	1	0	0	0	0	0	0	0	0	0	0	0	0	0	0	4	13
Theatre setup and equipment needed	0	0	0	1	1	0	0	0	0	1	0	0	0	0	0	1	1	0	0	0	1	0	0	1	0	1	0	0	0	0	0	8	26
Patient, surgeon and trocar positions	0	1	0	1	1	0	1	0	1	1	1	0	0	0	0	1	1	0	0	0	1	0	0	1	0	1	0	0	0	1	1	14	45
Anatomic demonstration	1	1	1	1	1	1	1	1	1	1	1	1	1	1	1	1	1	1	1	0	1	0	1	1	1	1	0	1	1	0	0	26	84
Step-by-step approach	0	0	0	1	1	0	1	0	1	1	1	0	0	0	0	1	1	0	0	0	1	0	0	1	0	1	0	0	0	0	0	11	35
Time in theatre and in hospital	0	0	0	0	0	0	0	0	0	0	0	0	0	0	0	0	1	0	0	0	0	0	0	1	0	0	0	0	0	0	0	2	6
Morbidity	0	0	0	0	0	0	0	0	1	1	0	0	0	0	0	1	1	0	0	0	0	1	0	1	0	0	0	0	0	0	0	6	19
Pictures of wounds and specimens	0	0	0	0	0	0	0	0	1	1	0	0	0	0	0	0	0	0	0	0	0	0	0	0	0	0	0	0	0	0	0		
Functional outcomes	0	0	0	0	0	0	0	0	1	1	0	0	0	0	0	1	0	0	0	0	0	1	0	1	0	1	0	0	0	0	0	6	19
Pictures, snapshots, diagrams and tables	0	0	0	1	0	0	0	0	1	1	0	0	0	0	0	1	1	0	0	0	0	0	0	0	0	0	0	0	0	0	0	5	16
Audio/Written comment	AW	None	None	A	A	None	None	W	AW	AW	A	A	None	W	None	AW	AW	None	None	None	A	A	None	A	None	A	None	None	None	None	None		
Total	4	4	2	8	8	3	5	4	12	14	6	3	3	4	3	13	11	2	2	2	8	4	4	11	4	8	2	4	4	3	3		
% of conformity	27	27	13	53	53	20	33	27	80	93	40	20	20	27	20	87	73	13	13	13	53	27	27	73	27	53	13	27	27	20	20		

A/W, Audio/Written; A, Audio; W, Written; N, None.

## Results

The first 50 videos were analyzed. Nineteen videos did not meet our criteria and were excluded. The mean number of views of the remaining 31 videos was 4,523 (range 37–24,086). The oldest video was uploaded in January 2000, and the most recent in November 2020. Duration ranged from 19 s to 22 min. The mean number of likes was 14.8 (range 0–80). The mean number of dislikes was 2.5 (range 0–14). The mean number of comments was 8.5 (range 0–67) (Table 1). The great part (82%) was posted by medical physicians. No one video contained all the principal steps of the procedure according (Table 2). A review of educational content revealed that 51% of videos did not contain either audio or written content. The audio explanation was present in 26% of the videos. The written content was present in 6% of videos. Both audio and written content was present in 17% of videos (Table 2). No video contained all 16 points of the LAP-VEGaS essential checklist (mean 5.4 points, range 2–14 points), with almost all neglecting preoperative information and outcomes (Table 2). The mean percentage of conformity was 36% (range 13%–93%). The most viewed videos were not associated with higher conformity to LAP-VEGaS guidelines showing only 4/16 points (25%). Title, the anonymity of the patients and anatomic demonstration were the most represented points while operating time, outcome and imaging was the most neglected aspect (Table 2).

## Discussion

The use of video-based learning is increasing in every aspect of life, especially in the field of medical education. YouTube® represents a more recognized platform for retrieving medical and surgical videos (9, 10).

YouTube®, to date, is the most used source of visual information about medical and surgical topics (11). It is a repository of thousands of surgical, animated, oral presentations and patient-experience videos. Many investigators, in the past years, evaluated the quality of YouTube videos addressing several medical and surgical topics (12). Because of the complete absence of the peer-review process, the assessment of the quality of the content is very important if these videos are being used for the education of residents or by experienced surgeons that aim to improve their skills or to perform a surgical procedure for the first time (13).

The open-access nature of YouTube® and the absence of a peer-review process, often lead to the poor quality of posted videos (11). The educational value of YouTube® remains undebatable and cannot be ignored. The LAP-VEGaS guidelines have been created by surgeons from multiple specialties with the intent to improve the educational value of videos used for training (10). The “ideal” video for educational purposes should include all the critical points addressing the checklist for the LAP-VEGaS guidelines, as well as the critical portions of the surgical procedure. This suggests that videos not responding to these criteria could be potentially misleading, providing unreliable data that may misinform the procedure (9, 12, 13). Video-assisted thoracoscopic sympathectomy is the most common surgical procedure performed for the treatment of PPH. Available videos often lack important domains of the procedure, do not cite sources, and demonstrate low conformity to LAP-VEGaS guidelines. The presence of educational videos on the platforms such as YouTube® is an advantage for the future of online learning but is not without consequences. The open-access nature of these platforms for video-sharing leads to a presence of unregulated and unstandardized methods which do not meet professional learning standards. Other online video repositories are present on the internet, and some are dedicated to surgical education. However, their quality has not consistently been shown to be superior to that found on YouTube® (10). One of the limitations of this study is that YouTube® searching was performed using the default settings which can vary by geographical location. The second limitation is that the search terms we used would be considered limited and may have potentially narrowed or excluded other relevant videos/results.

## Conclusions

Our study demonstrates that videos addressing VATS available on YouTube® did not shows high quality when applying the LAP-VEGaS criteria. Available videos often lack important steps of the procedure, do not cite sources, and show low conformity to LAP-VEGaS guidelines. Medical professionals should consider this when using it for educational purposes in their routine clinical practice.

## Data Availability

The original contributions presented in the study are included in the article/Supplementary Material, further inquiries can be directed to the corresponding author.
